# Production of thyrotropin receptor antibodies in acute phase of infectious mononucleosis due to Epstein–Barr virus primary infection: a case report of a child

**DOI:** 10.1186/s40064-015-1236-8

**Published:** 2015-08-27

**Authors:** Keiko Nagata, Keisuke Okuno, Marika Ochi, Keisuke Kumata, Hitoshi Sano, Naohiro Yoneda, Jun-ichi Ueyama, Michiko Matsushita, Satoshi Kuwamoto, Masako Kato, Ichiro Murakami, Susumu Kanzaki, Kazuhiko Hayashi

**Affiliations:** Division of Molecular Pathology, Department of Pathology, Faculty of Medicine, Tottori University, 86 Nishi-cho, Yonago, Tottori 683-8503 Japan; Division of Pediatrics and Perinatology, Department of Multidisciplinary Internal Medicine, Tottori University, 36-1 Nishi-cho, Yonago, Tottori 683-8504 Japan

**Keywords:** Epstein–Barr virus (EBV), Infectious mononucleosis, Lytic infection, Reactivation, Autoantibody, Thyrotropin receptor antibody (TRAb), Graves’ disease

## Abstract

Various autoantibodies have been reported to be detected during the progression of infectious mononucleosis. We observed a case of infectious mononucleosis due to Epstein–Barr virus primary infection for 2 months, and noticed the transiently increased titer of thyrotropin receptor autoantibodies detected at the acute phase on the 3rd day after admission. At that time, real-time quantitative PCR also revealed the mRNA expressions of an immediate early lytic gene, *BZLF1*, and a latent gene, *EBNA2*. The expression of *BZLF1* mRNA means that Epstein–Barr virus infects lytically, and EBNA2 protein has an important role in antibody production as well as the establishment of Epstein–Barr virus latency. These results suggest that Epstein–Barr virus lytic infection is relevant to thyrotropin receptor autoantibody production. Thyrotropin receptor autoantibodies stimulate thyroid follicular cells to produce excessive thyroid hormones and cause Graves’ disease. Recently, we reported the thyrotropin receptor autoantibody production from thyrotropin receptor autoantibody-predisposed Epstein–Barr virus-infected B cells by the induction of Epstein–Barr virus lytic infection in vitro. This case showed in vivo findings consistent with our previous reports, and is important to consider the pathophysiology of Graves’ disease and one of the mechanisms of autoimmunity.

## Background

Various autoantibodies have been reported to be detected in the serum, during the clinical course of infectious mononucleosis (IM), which can be one of the factors explaining the relevance of Epstein–Barr virus (EBV) to autoimmune diseases (Sutton et al. 1974; Longnecker et al. 2013).

EBV is a common virus of Herpesviridae, and over 90 % of adults have its serum antibody. However, in developed countries, 50 % of children and young adults are seronegative and primary infection even in infants and young children is usually symptomatic or has nonspecific symptoms (Longnecker et al. 2013).

Since a report by Henle et al. (1974), the serum levels of EBV antibodies from onset to the convalescent or chronic phase have been well investigated (Longnecker et al. 2013; Henle et al. 1974; Cohen 2006; Luzuriaga and Sullivan 2010). Recently, real-time PCR for EBV viral load has become available, as well as serum titers of EBV antibodies for diagnosis. However, there are few reports monitoring the expression of EBV latent gene mRNA and lytic gene mRNA. We have been studying EBV reactivation (lytic infection) and autoimmune hyperthyroidism, and reported that EBV lytic infection induction in vitro causes the production of thyrotropin (TSH) receptor antibodies (TRAbs) from host TRAb-predisposed EBV-infected (TRAb(+)EBV(+)) B cells (Nagata et al. 2014, 2015).

We diagnosed this case of IM, as an EBV primary infection and followed it up for 2 months. Blood sampling was performed three times, once in the acute phase and the others in the convalescent phase. Investigation of these samples revealed that mRNA of an immediate early lytic gene, *BZLF1*, and of a latent gene, *EBNA2*, was expressed in the acute phase, and that heterogeneous autoantibodies against TSH receptor, TRAbs, were also detected in the same acute lytic phase.

This case is important for considering the mechanisms of autoantibody production induced by EBV lytic infection.

## Case description

A boy who was 3 years and 10 months old was admitted to our hospital on July 23. He had fever, appetite loss, and bilateral cervical lymph node enlargement with pain from July 21. We noticed that the tonsils were swollen with white exudates, the infra-auricular lymph nodes (right 5 × 2 cm, left 4 × 2 cm) were swollen and painful, and several neck lymph nodes (1 cm) were palpable. Neither axillar nor inguinal lymph nodes were swollen. The abdominal region was soft and flat, and hepato-splenomegaly was not apparent.

**Fig. 1:**
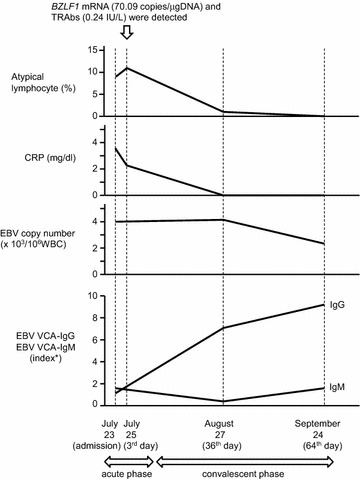
Time-course change in this case. According to the decrease of inflammation represented by CRP, the number of atypical lymphocytes decreased. The copy number of EBV began to decline in the convalescent phase. In the acute phase, EBV VCA-IgM was higher than EBV VCA-IgG, but in the convalescent phase, EBV VCA-IgG was higher. *BZLF1* mRNA (70.09 copies/μgDNA) and TRAbs (0.24 IU/l) were detected in the acute phase. *Index stands for sample absorbance/absorbance of cut-off serum. *EBV* Epstein–Barr virus, *VCA* viral capsid antigen, *BZLF1* one of the EBV-immediate-early lytic genes, *TRAb* thyrotropin receptor antibody

Laboratory data are shown in Table 1 and Fig. 1. C-reactive protein (CRP) increased to 3.63 mg/dl on the day of admission, but granulocytes were not increased. Lymphocytes increased to 50 %, with 9 % atypical lymphocytes. The patient was anemic and had slight liver dysfunction. Negative cytomegalovirus (CMV)-IgM and high titer of CMV-IgG indicated past infection of CMV, and positive EBV VCA-IgM was consistent with IM due to EBV primary infection.

**Table 1:** Laboratory data

	July 23	July 25	August 27	September 24
	Admission	3rd day	36th day	64th day
RBC (/μl)	3.86 × 10^6^	3.71 × 10^6^	4.24 × 10^6^	4.18 × 10^6^
Hgb (g/dl)	10.9	10.7	11.6	11.3
Platelet (/μl)	220 × 10^3^	231 × 10^3^	259 × 10^3^	239 × 10^3^
WBC (/μl)	9800	12,400	4600	5600
Segmented (%)	32	15	41	38
Band (%)	0	2	0	0
Lymphocytes (%)	50	64	54	57
Monocytes	9	8	3	2
Atypical lymphocytes (%)	9	11	1	0
Reticulocyte (%)	3.8	3.1	1.3	1.2
Total bilirubin (mg/dl)	0.4		0.5	0.4
AST (IU/l)	35	36	32	31
ALT (IU/l)	23	24	20	19
LDH (IU/l)	537	567	260	268
CRP (mg/dl)	3.63	2.2	0.02	0.02

Except for antibiotics against *Moraxella Catarrhalis* 4+ and *Haemophilus Influenzae* 3+ detected from throat swab culture, rest alone was effective for recovery and the patient left hospital on the 5th day. He was observed for 2 months as an outpatient.

From the blood samples, one taken in the acute phase and two in convalescent phase, we measured serum titers of EBV-VCA, -EBNA, -EA-D, and total immunoglobulin by ELISA and EBV copy numbers by real-time PCR (Kimura et al. 1999).

In the examination on the 36th day, the condition of the patient was good, and the serum data indicated that he was already in the convalescent phase, with a decrease of VCA-IgM and an increase of VCA-IgG. EBV copy number was still high, but it usually remains high for 6 weeks after onset, so we considered that this would be reasonable given the development of immunity in this child (Longnecker et al. 2013). Through the follow-up, total immunoglobulin changed almost in parallel with VCA antibodies.

We examined serum autoantibodies of the 3 stored samples (at day 3, 36, and 64): anti-nuclear antibody (ANA) and anti-smooth muscle antibody (SMA), using fluorescent antibody (FA), and TRAbs by radio-receptor assay, at the same time for each antibody (Table 2). TRAb radio-receptor assay (DYNOtest TRAb Human; Yamasa Corporation, Choshi, Japan) were performed according to manufacturer’s instruction (clinical cut-off is 1 IU/l).

**Table 2:** EBV copy numbers, serum antibodies, and expression of EBV mRNA

	July 25	August 27	September 24
	3rd day	36th day	64th day
EBV copy number (/10^6^ WBC)	4.1 × 10^3^	4.2 × 10^3^	2.6 × 10^3^
EBV-EA-IgG (index*)	0.4 (−)	0.3 (−)	0.3 (−)
EBV-EBNA-IgG (index*)	0.1 (−)	0.2 (−)	1.9 (+)
EBV-VCA-IgM (index*)	1.5 (+)	0.6 (±)	1.4 (+)
EBV-VCA-IgG (index*)	1.1 (+)	6.6 (+)	8.9 (+)
Total-IgG (mg/ml)	11.15	13.37	9.49
Total-IgM (μg/ml)	836.2	815.05	825.1
Autoantibodies			
ANA (x)	(−)	(−)	(−)
SMA (x)	(−)	(−)	(−)
TRAbs (IU/l)	0.24	(−)	(−)
EBV mRNAs (copies/μgDNA)			
*LMP1*	(−)	(−)	(−)
*LMP2*	(−)	(−)	(−)
*EBNA1*	(−)	(−)	(−)
*EBNA2*	4.29	(−)	(−)
*BZLF1*	70.09	(−)	(−)
*EA*-*D*	(−)	(−)	(−)

Each value of mRNA is normalized to the expression of β-actin

We used EBV mRNA from B95-8 strain as a reference

*EBV* Epstein–Barr virus, *EA* early antigen, *EBNA*, Epstein–Barr nuclear antigen, *VCA* viral capsid antigen, *LMP* latent membrane protein, *TRAbs* thyrotropin receptor antibodies, *SMA* smooth muscle antibody, *ANA* antinuclear antibody

* Index stands for sample absorbance/absorbance of cut-off serum

We constructed primers and probes for EBV latent genes and lytic genes (Table 3); then, we performed real-time quantitative PCR to detect the mRNA expression of EBV latent genes and lytic genes (Table 2) (Kubota et al. 2008; Ryan et al. 2004). The results indicated that mRNA of an immediate early lytic gene, *BZLF1*, and a latent gene, *EBNA2*, was detected in the acute phase, and that TRAbs were also detected in the same acute phase.

**Table 3:** Primers and probes for real-time quantitative PCR of EBV

Assay	Sequence	References
LMP1		
Sense	5′-CCC TTT GTA TAC TCC TAC TGA TGA TCA C	Kubota et al. (2008)
Antisense	5′-ACC CGA AGA TGA ACA GCA CAA T	Kubota et al. (2008)
Probe	5′-CTC ATC GCT CTC TGG AAT TTG CAC GG	Kubota et al. (2008)
LMP2		
Sense	5′-AGC TGT AAC TGT GGT TTC CAT GAC	
Antisense	5′-GCC CCC TGG CGA AGA G	
Probe	5′-CTG CTG CTA CTG GCT TTC GTC CTC TGG	
EBNA1		
Sense	5′-TAC AGG ACC TGG AAA TGG CC	Ryan et al. (2004)
Antisense	5′-TCT TTG AGG TCC ACT GCC G	Ryan et al. (2004)
Probe	5′-AGG GAG ACA CAT CTG GAC CAG AAG GC	
EBNA2		
Sense	5′-TCT TGC GTT ACA TGG GGG AC	
Antisense	5′-CCT GGT AGG GAT TCG AGG GA	
Probe	5′-AAT TGT TGA CAC GGA TAG TCT TGG	
BZLF1		
Sense	5′-AAA TTT AAG AGA TCC TCG TGT AAA ACA TC	Ryan et al. (2004)
Antisense	5′-CGC CTC CTG TTG AAG CAG AT	Ryan et al. (2004)
Probe	5′-ATA ATG GAG TCA ACA TCC AGG CTT GGG C	
EA-D		
Sense	5′-CGT GCC AAT CTT GAG GTT TT	
Antisense	5′-CAC CCG GGG ACT TTT ATC TT	
Probe	5′-TTT ATT TAA CCA CGC CTC CG	
β-Actin		
Sense	5′-CCT GGC ACC CAG CAC AAT G	
Antisense	5′-GCC GAT CCA CAC GGA GTA CT	
Probe	5′-ATC AAG ATC ATT GCT CCT CCT GAG CGC	

As a complication, this case had coagulopathy due to deficit of von Willebrand factor, but this disease is not accompanied by immune disorder and rarely has an effect on viral infection.

## Discussion and evaluation

In EBV primary infection, most B cells become latently infected lymphoblastoid cells, and some B cells become lytic infected cells (Longnecker et al. 2013; Cohen 2000). The lymphocytes of this case expressed mRNA of *BZLF1*, the immediate early gene of EBV lytic infection, and mRNA of *EBNA2*, the latent gene, in the acute phase (Table 2; Fig. 1). The expression of *BZLF1* mRNA indicates that EBV lytic infection occurred in the acute phase of IM. EBNA2 is important for B cell transformation when EBV establishes its latent infection and is the transactivator of various genes, including LMP1 that activates NF-κB (Longnecker et al. 2013); thus, it is related to antibody production and the expression of activation-induced cytidine deaminase (Tran et al. 2010).

During IM, mRNA for latent genes as well as lytic genes could be expressed, but in this case, we could not detect mRNA other than *BZLF1* and *EBNA2*. This might be related to the probably low potential of the immune system of this case of 3-year-old child to react to the invasion of viruses (Longnecker et al. 2013; Piątosa et al. 2010; Parham 2009). Children at this age are also known to have weak antibody production (Longnecker et al. 2013; Parham 2009). In this case, we could not detect EA-D serum antibody and EA-D mRNA.

The levels of EBV VCA-IgM and serum total IgM were high in the acute phase and those of EBV VCA-IgG and serum total IgG rose in the convalescent phase (Table 2). These results imply that IgM production may be caused by EBV acute infection (Nakamura et al. 1988; Casali et al. 1990).

Sutton et al. (1974) showed that SMA was detected at the onset of IM and declined in the convalescent phase, and that ANA and rheumatoid factor (RF) are rarely present and do not preferentially appear in the acute phase (Sutton et al. 1974; Longnecker et al. 2013). These increases of antibody production are considered to be the result of polyclonal B-cell activation in IM (Longnecker et al. 2013).

In our case, ANA and SMA were negative throughout the observation period, but we could detect TRAbs at a low titer (Table 2). FA tests for ANA and SMA are common, but not sensitive compared with TRAb radio-receptor assay. Using a more sensitive ELISA system for ANA and SMA, a certain amount of antibodies might be detected, despite a low titer.

Several viral infections are recognized to induce autoantibody production, but the antibodies are often low level, and do not develop any clinical symptoms (Mandel et al. 2011; Poole et al. 2011; Camarero et al. 2008). Our case is too young to produce sufficient antibodies, and did not show any specific autoimmune symptoms.

However, there are some cases reported to have developed Graves’ disease related to IM (Akahori et al. 2010), and this child needs long-term follow-up.

We observed that EBV lytic infection and autoantibody production occurred in the same period, which suggests that EBV lytic infection stimulates autoantibody production.

EBV is a latent virus and persists mainly in B lymphocytes. B cells differentiate into antibody-producing cells; thus, persistent EBV may stimulate the antibody production of host cells (Nagata et al. 2011, 2014, 2015). We have been investigating TRAb-production in Graves’ disease induced by EBV reactivation (lytic infection) (Nagata et al. 2011, 2014, 2015). Our hypothesis is that lytic change of EBV in TRAb-predisposed and EBV-infected (TRAb(+)EBV(+)) cells would stimulate host B cells to promote TRAb production with different level of efficiency between patients and controls, and may cause the development or exacerbation of Graves’ disease. We showed that TRAb(+)EBV(+) cells really exist in the peripheral blood of Graves’ disease patients and healthy controls (Nagata et al. 2014), and these cells released TRAbs in culture fluid when we induced lytic change in persistent EBV (Nagata et al. 2015).

In EBV primary infection, some B cells become lytic (Longnecker et al. 2013; Cohen 2000). According to our hypothesis, these lytic infected B cells with TRAb predisposition, differentiate to plasma cells and produce TRAbs. The result of research of this case in which EBV lytic gene expression and autoantibody production occurred simultaneously in the acute phase is consistent with our previous data in vitro and provides a suggestive example in vivo.

## Conclusions

We have reported a 3-year-old patient with IM due to EBV primary infection. This case simultaneously showed EBV lytic gene expression and autoantibody (TRAb) production in the acute phase of the disease.

This case is important to consider the pathophysiology of Graves’ disease and the mechanisms of autoantibody production induced by EBV lytic infection.

## Abbreviations

EBV: Epstein–Barr virus
IM: infectious mononucleosis
TSH: thyrotropin
TRAb: thyrotropin receptor antibody
CMV: cytomegalovirus
EA: early antigen
EBNA: Epstein–Barr nuclear antigen
VCA: viral capsid antigen
LMP: latent membrane protein
ANA: antinuclear antibody
SMA: smooth muscle antibody
FA: fluorescent antibody
RF: rheumatoid factor
MS: multiple sclerosis
SLE: systemic lupus erythematosus
